# Exploring environmental coverages of species: a new variable contribution estimation methodology for rulesets from the genetic algorithm for rule-set prediction

**DOI:** 10.7717/peerj.8968

**Published:** 2020-05-12

**Authors:** Anni Yang, Juan Pablo Gomez, Jason K. Blackburn

**Affiliations:** 1 Spatial Epidemiology & Ecology Research Laboratory, Department of Geography, University of Florida, Gainesville, FL, USA; 2 Emerging Pathogens Institute, University of Florida, Gainesville, FL, USA; 3 Departamento de Química y Biología, Universidad del Norte, Barranquilla, Colombia

**Keywords:** Variable selection, GARP, Physiological mechanisms, Prevalence, Median range, Ecological niche modeling, Spatial prediction, Variable contribution, *Toxostoma rufum*

## Abstract

Variable contribution estimation for, and determination of variable importance within, ecological niche models (ENMs) remain an important area of research with continuing challenges. Most ENM algorithms provide normally exhaustive searches through variable space; however, selecting variables to include in models is a first challenge. The estimation of the explanatory power of variables and the selection of the most appropriate variable set within models can be a second challenge. Although some ENMs incorporate the variable selection rubric inside the algorithms, there is no integrated rubric to evaluate the variable importance in the Genetic Algorithm for Ruleset Production (GARP). Here, we designed a novel variable selection methodology based on the rulesets generated from a GARP experiment. The importance of the variables in a GARP experiment can be estimated based on the consideration of the prevalence of each environmental variable in the dominant presence rules of the best subset of models and its coverage. We tested the performance of this variable selection method based on simulated species with both weak and strong responses to simulated environmental covariates. The variable selection method generally performed well during the simulations with over 2/3 of the trials correctly identifying most covariates. We then predict the distribution of *Toxostoma rufum* (a bird with a cosmopolitan distribution) in the continental United States (US) and apply our variable selection procedure as a real-world example. We found that the distribution of *T. rufum* could be accurately modeled with 13 or 10 of 21 variables, using an UI cutoff of 0.5 or 0.25, respectively, arriving at parsimonious environmental coverages with good model accuracy. We also provide tools to simulate species distributions for testing ENM approaches using R.

## Introduction

Ecological niche models (ENMs) have been widely applied in ecology, biogeography, conservation biology, evolution, and epidemiology over the past several decades (Peterson & Vieglais, 2001; Pearson & Dawson, 2003; Ostfeld, Glass & Keesing, 2005; Larson et al., 2010). Modeling a species’ ecological niche and geographic distribution relies on some form of pattern-recognition based on non-random association between the geographic occurrences of a species and environmental conditions that support its survival under the ecological niche theory (Hutchinson, 1957; Araujo & Guisan, 2006). The ecological niche of a species can be defined as the environmental conditions that allow the population to be maintained without immigration (Grinnell, 1917; Pulliam, 1988) and can be described by an n-dimensional hyper-volume of environmental covariates that determine the ecological space of the species (Hutchinson, 1957). Hence, the accuracy of predicted distributions is primarily driven by the adequacy of environmental covariates used in the models (Araujo & Guisan, 2006; Austin, 2007). Species’ distributions and their environmental requirements can be veiled or misleading due to the selection of inappropriate predictors (Araujo & Guisan, 2006). Incorporating the suitable covariates in ecological niche modeling experiments remains an important area of research with continuing challenges.

Most ENM algorithms use exhaustive searches through environmental variable space (in multiple combinations) in order to identify the environmental covariates that define a species’ distribution. As the most biologically-based decision in ENMs, the selection of environmental covariates should primarily depend on the knowledge of the adaption of species’ physiology to the ecological or biological conditions (ecophysiological or biophysiological processes) that govern the relationships between a species and the environment (Austin, 2007). However, this information is difficult to obtain in many cases, especially for some poorly understood species. With a large number of potential predictors, including biotic and abiotic, direct and indirect factors, which influence species’ responses to environmental gradients and available resources (Austin & Van Niel, 2011), some crucial questions arise, like “how many variables are enough” and “which variables need to be included” (Huston, 2002; Araujo & Guisan, 2006). The evaluation of variable contributions within ENMs is an alternative to quantify the relationship between the species survival and environment to understand the ecological requirements of a species. The estimation of variable contribution in the ENMs provides an objective metric to infer the strength of species response to the environmental conditions, which can help to hypothesize about the ecophysiological processes determining the geographical distributions and understand some basic biology of the species (Araujo & Guisan, 2006). Finally, the environmental covariates contributing most are selected to interpret the species’ ecological niche and predict the most likely distribution (species range).

The estimation of each variable’s explanatory power and the selection of the optimal variable set within models, however, can be challenging for some ecological niche modeling approaches, such as the Genetic Algorithm for Ruleset Production (GARP). GARP predicts species distributions based on presence-only data via an algorithm employing a superset of logistic regression, range and negated range rules, and atomic (bioclim) rules (Stockwell, 1999). GARP is one of the 13 key ENMs and software available to the species distribution modeling community (Ahmed et al., 2015). Although several algorithms and advanced methods have been introduced, and many, like MaxEnt, frequently used in the literature, GARP is still widely used to model species distribution and understand their ecological affinities. Some recent applications include predicting distributions of different species, such as the invasive species (e.g., pignut in India Padalia, Srivastava & Kushwaha, 2014 and creeping oxeye in Central America, Qin et al., 2015), modeling bird abundance patterns (Martínez-Meyer et al., 2013), endangered bird species (Montenegro et al., 2017), and ecological niche of tree species (Prakash Singh et al., 2013), and delineating disease risk areas by estimating the geographical distribution of pathogens (Barro et al., 2016; Chikerema et al., 2017) and vector species (Ramsey et al., 2015; Sloyer et al., 2018; Lippi et al., 2019). Other research compares GARP with some other ENMs (especially MaxEnt) to show how species distributions change using different approaches to provide reliable predictions (Padalia, Srivastava & Kushwaha, 2014; Wang et al., 2017; Ray, Behera & Jacob, 2018), to compare the predictive performance of different methods (Khatchikian et al., 2011; Zhu & Peterson, 2017), or to understand why the differences in the performance exist (Elith & Graham, 2009). Therefore, it is of primary importance to revisit GARP and better understand what biological information can be obtained from rule-set development during the modeling process. Specifically, it is important to determine best practices for how rule-sets can be mined to examine covariate contributions to species’ distribution predictions.

Genetic Algorithm for Ruleset Production experiments can employ the Jackknife procedure (Peterson & Cohoon, 1999; Levine et al., 2007; Thomasson & Blouin-Demers, 2015), but there is no easy way and rubric for the estimation of variable contribution. While the selection of the procedure is straight forward, the interpretation has not been standardized or widely used. Levine et al. (2009) presented a method for performing a statistically based between the comprehensive map (i.e., *N* variables) and jackknifed maps (i.e., *N −* 1 variables) generated from GARP to determine the optimal ecological parameters for predicting human monkeypox disease. The larger differences found between the output from an experiment with all variables and the map produced from a jackknifed experiment, the greater the contribution the reduced variable made in those experiments (Levine et al., 2009). However, this estimation relies on the prediction performance of GARP and assumes that the comprehensive map, as the base map, represents the geographic distribution predicted by the “true” fundamental niche. Also, the computational intensity for massive iterations of the jackknife procedure makes variable selection difficult when there is a large set of potential environmental covariates. Alternatively, Sweeney, Beebe & Cooper (2007) employed an external classification and regression tree (CART) to select the optimal environmental layers to be used in GARP experiments to model the distribution of *Anopheles punctulatus* in Australia. However, GARP and CART use different algorithms to determine relationships between species’ occurrences and environmental covariates. GARP includes logistic regression and range envelopes, while CART constructs decision trees by making binary splits of the covariates. These differences in algorithms may result in different estimations of variable explanatory power and therefore the variable set selected by CART may not be optimal for GARP.

Exploring the environmental space that defines the ecological niche of a species can help us in understanding the underlying ecophysiological processes of the species’ distribution. Thus, it is of primary importance to develop tools for ENMs that explore variable space or rubrics to assess variable contributions. Here, we present a novel variable contribution estimation methodology for GARP based on the exploration of the GARP rulesets to consider the explanatory power of variables within a modeling experiment and the biological information within the experiment using those variables. We base our variable selection process mainly in two metrics: (1) the prevalence of each environmental variable in the dominant presence rules of the best model subset from a GARP experiment, and (2) the variables’ median range in those rules. In this study, we explain in detail the new variable contribution estimation procedures and test its performance using simulations and provide a real-world case study for exploring ecological requirements and predicting the distribution of the brown thrasher, *Toxostoma rufum*, a cosmopolitan bird species in the continental US using a bioclimatic variable set recently introduced to the modeling community.

## Materials and Methods

### Genetic Algorithm for Ruleset Production

**Figure 1:**
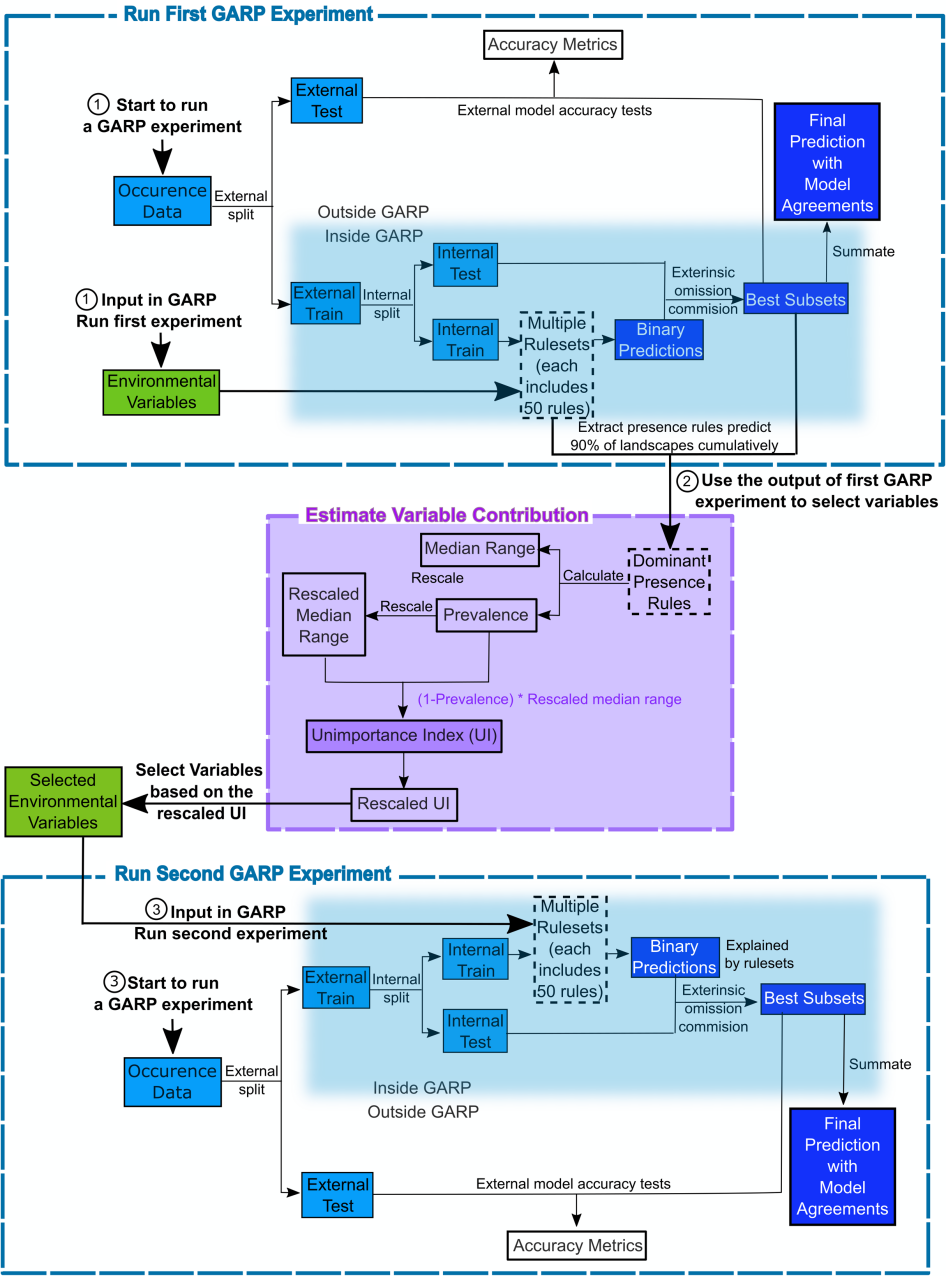
Flowchart depicting the procedure to run a GARP experiment and estimate variable contribution. There are three steps for predicting species distribution and selecting variables selected via Unimportance Index (UI). First, run a complete GARP experiment with the full variable set. Second, use the output of the first GARP experiment to rank and select variables based on UI. Third, input the important variables in GARP to run the second GARP experiment to predict the species distributions.

The Genetic Algorithm for Ruleset Production is a presence-only iterative modeling algorithm that searches for non-random relationships between point occurrence data and environmental covariates. For this study, we use DesktopGARP (DG) version 1.13 to perform GARP experiments. Readers can freely access this version of DG from GitHub (https://github.com/jkblackburn/DesktopGARP1.1.3). The procedure for running a GARP experiment is demonstrated in Fig. 1. Initially, we split the occurrence data into external training and testing sets. The external training set is inputted in DG for model building, while the testing set is withheld for external model accuracy tests to evaluate the performance of GARP experiment. Each properly executed GARP experiment will include multiple models and each will have a ruleset with 50 rules predicting presence or absence (note: there are GARP implementations in openModeller allowing the user to control the number of rules). There are four types of rules (range, negated range, atomic, or logit) described as the if/then logic statements. Range rules specify the envelope with upper and lower bounds for the presence of the species (e.g., IF temperature = (10.2–13.5 °C) AND NDVI = (0.15–0.23) THEN species = PRESENCE). Negated range rules define the conditions outside of variable ranges (e.g., IF NOT temperature = (10.2–13.5 °C) AND NDVI = (0.15–0.23) THEN species = ABSENCE). Logit rules employ logistic regression to determine the relationship between the species occurrence and covariates (e.g., IF temperature × 0.0037 + NDVI × 0.57 THEN species = PRESENCE). The presence or absence of the species in the logit rule type is determined based on the probability of the occurrence of the species predicted by the logistic regression with the threshold of 0.5. Atomic rules use specific values of the covariates to determine the presence of the species (e.g., IF temperature = 12.5 °C AND NDVI = 0.19 THEN species = PRESENCE). Those rules are developed and tested internally using random draws of presence points from the known occurrences and random draws of the background space representing absences (i.e., pseudo-absences). An internal chi-square test built on the predicted and observed values is used to evaluate the quality of each rule at predicting presence or absence with the user’s pre-defined proportion of input data (internal testing set). GARP can accept, modify or delete rules using deletions, insertions, cross-overs, among other types of mutations to improve predictive accuracy in a genetic fashion.

Once a ruleset is developed, it is projected onto the geography of the study area to develop a presence/absence map describing the species’ potential geographic distribution, for example, Blackburn (2006), Joyner (2010) and Stockwell (1999). Given the iterative nature of GARP, the model does not arrive at a single solution. DG splits input occurrence data into training and testing sets inside the software for model evaluation and incorporates a “best subset” procedure, which would select the best subset of models based on two criteria: omission (false negative) and commission (false positive; percent of pixels predicted present) rates. Such calculations are performed on each individual model and the “best subset” procedure selects a user defined number of models based on specific omission and commission values. Here, experiments were setup to run up to 200 models, we selected 20 models with no more than 10% “extrinsic” omission rate, which is calculated from the internal testing set. A median commission percentage is then calculated for the 20 low-omission models. Investigators can define the percentage (defaulted to 50%; 10 models) of the low-omission models that have individual commission closest to the median to be selected as the best subset (McNyset & Blackburn, 2006). Finally, the best subset with 10 best presence-absence predictions can be summed and mapped on the landscape with model agreements indicating the likelihood of the species presences. GARP has been shown to perform well across the spectrum of species’ prevalence on the landscape from rare to common making it useful for management oriented studies focused on relating geographic potential to management or conservation needs (Peterson, Papeş & Eaton, 2007). A more extensive description of GARP’s modeling framework and test of its performance can be found elsewhere (Anderson, Lew & Peterson, 2003; Martinez-Meyer et al., 2006; Peterson & Cohoon, 1999; Stockwell, 1999), and in this study, we limit our objectives to describe the variable selection procedure.

### Conceptual framework for variable contribution estimation procedures

We designed a new variable selection methodology to estimate variable contributions to species distributions in GARP. We used accuracy metrics (omission and commission rates and area under the curve (AUC)) to select the best subset of models (rulesets) in the GARP experiment. We measured the variable contributions based on two criteria: (1) the prevalence of the variable in the dominant presence rules and (2) the scaled median range for those variables across the rules within the best subset of the GARP experiment.

The prevalence of a variable in the dominant presence rules of the best subset is defined as the frequency with which the variable predicts the presence of the species in the dominant presence rules of the best subset (See Eq. (1)). With the best subset process activated, DG selects a set of best models as described above. The dominant presence rules in the best subset are defined as a subset of rules that cumulatively predict the over 90% of the species’ presence on the landscape in the top-selected 10-model subset (Mullins et al., 2011). Those rules represent the primary suitable environmental conditions that define the core of the ecological niche of the species (based on the set of variables available) but does not take into account rare situations in which species are occasionally or temporarily present. Here we only analyzed presence rules, since absence rules tend to have wide median ranges. We defined prevalence as:

(1) $$\eqalign{ & {\rm Prevalence}_{({\rm best\ subset})} \cr & =\frac{\rm the\;number\;of\;times\;the\;variable\;is\;present\;in\;the\;dominant\;presence\;rules}{\rm total\; number\; of\; dominant\; presence\; rules}}$$

The high prevalence rate of a variable indicates that the variable is frequently used to predict the presence of the species in the best subset. Thus, a variable with a higher prevalence rate suggests the variable is relatively more important in the GARP experiment.

The median range of a variable is defined as the difference between the median values from a set of upper bound and lower bound (i.e., maximum and minimum values) of this variable (See Eq. (2)) in the dominant presence rules from the best subset (Joyner, 2010). For different types of rules, the maximum and minimum values are extracted in different ways. In range and negated range rules, the maximum and minimum values are extracted directly from the upper and lower boundaries recorded in the rulesets. For the logit rules, the maximum and minimum values are extracted from the landscape where those logit rules are used to predict the presence of the species via zonal statistics. For atomic rules, the specific values of the covariates that predict the presence of the species are directly extracted from the rules. We then compare the extracted value of the atomic rules with the maximum and minimum values from other types of rules to evaluate whether it fell inside the coverage. To quantitatively compare the median ranges of different variables, we scale the median range of each variable from 0 to 1 (Barro et al., 2016). A variable with a wide median range indicates that the presence of species is not sensitive to this predictor, while a variable with a narrow median range suggests that the occurrence of the species is constrained to specific conditions regarding the covariate (Mullins et al., 2011; Barro et al., 2016).

(2) $${\rm Median\; range}_{\rm cov} = {\rm median\; maximum\; value}_{\rm cov} - {\rm median\; minimum\; value}_{\rm cov}$$

We measured the variable contribution to GARP based on an Unimportance Index (UI) to consider both criteria, the prevalence rate and scaled median range. The UI of each covariate is calculated as the multiplication of the scaled median range and the probability that the variable is not used to predict the presence of the species in the dominant presence rules of the best subset (Eq. (3)). This multiplication would help to combine and balance both criteria. Variables with less contribution to a GARP experiment are defined as the ones with wider median range and lower prevalence. Therefore, the larger the UI value is, the less contribution the associated variable brings to the model. To clearly compare and evaluate variable contribution we finally rescaled the UI to 0–1 following Eq. (4):

(3) $${\rm UI} = (1 - {\rm prevalence})* {\rm median\; range}$$

(4) $${\rm Rescaled \; UI}_k = {{\rm UI}_k - {\rm UI}_{\rm min}\over{{\rm UI}_{\rm max} - {\rm UI}_{\rm min}}}$$

where UI*_k_* is the unimportance index for covariate *k*; UI_max_ and UI_min_ are the maximum and minimum value of the UIs for the covariates in the variable set, respectively. This procedure of the estimation of variable contributions are shown in Fig. 1 and programed in “GARPTools” R-package (freely and immediately available at https://github.com/cghaase/GARPTools).

### Testing the performance of the new variable selection procedure using simulations

#### Simulating the species and sampling it

Although several previous studies have compared the predictive accuracies and performance of different algorithms used to model species’ ecological niches (Elith & Graham, 2009; Barbet-Massin et al., 2012), there is still no gold standard ecological niche modeling approach for evaluating the performance of the aforementioned variable contribution estimation method. To address this, we simulated species with known responses to the environment and geographical distributions and tested the number of covariates being correctly selected by our variable selection method. In this way, we can evaluate the performance of DG against ideal distributions rather than guessing at the ideal distribution of a species in the real world. The simulator is provided in R code in the Supplemental Files. We first generated ten normally distributed environmental covariates with spatial autocorrelation on a 10.5 × 10.5 degree landscape at a 0.01 degree resolution (Supplemental Information 1). Five of those covariates were simulated using an exponential variogram model with a range of 10, sill of 1, and nugget of 0, the others used a spherical variogram model with a range of 6, sill of 1, and nugget of 0. Next, we simulated 200 species using three variables from the entire set drawn at random without replacement. The probability of occurrence was computed as:

(5) $$P ( {\rm probability\; of\; occurrence} ) = {{\rm e}^{ - ( {{{( {{{\rm \beta} _1}{*}{x_1} + {{\rm \beta} _2}{*}{x_2} + {{\rm \beta} _3}{*}{x_3}})}^2}} )}}$$

where β_1_, β_2_ and β_3_ are the coefficient that determines the influence of each covariate on the species distribution and *x*_1_, *x*_2_ and *x*_3_ are the environmental covariates. The three selected variables used in species distribution simulation were recorded for further validation of the performance of the variable selection procedures. Once we obtained the probability surface on the landscape, we used it as the success probability of a Bernoulli random trial to obtain the true distribution (Elith & Leathwick, 2009). The three coefficients for each species were sampled from a normal distribution under two scenarios. The first represents a scenario in which the environmental covariates weakly define the species distribution. In this case, we sampled the coefficients from a normal distribution with mean of one and standard deviation of 0.5. For the second scenario we assumed that the coefficients had a stronger effect on the distribution of the species such that the coefficients were normally distributed with mean of five and a standard deviation of 0.5. We simulated 100 species using weak effect coefficients and 100 using strong effect. Finally, we randomly extracted 50 presence locations from the centroid of the grid cells of the realized distribution for each simulated species (binary presence–absence distribution) as the presence-only data to input in GARP.

#### Testing the variable selection performance

To test the performance of the UI, we used the full set of ten environmental variables and the 50 presence points sampled from the species distribution to generate a GARP experiment for each species. Here, since the true distributions of the simulated species is known, we can directly compare the predictions with true distributions without withholding part of data for external model validation. We set the training/testing data split to 75%/25% inside DG. To maximize GARP performance, model runs were set to a maximum of 1,000 iterations or until convergence of 0.01. The best subset procedure selected ten best models under a 10% extrinsic omission threshold and a 50% commission threshold (Fielding & Bell, 1997). Those 10-model best subsets were added together using the GARPTools R-package.

For each of the 200 species we calculated the UI for all the ten variables used in model development and recorded the three variables with the lowest UI (i.e., the three variables with highest contribution to the predicted distributions). We evaluated the performance of the model and the UI by counting the number of variables *r* (*r* = 0, 1, 2, 3) correctly identified by the model for each of the species. Next we counted the number of species *s* (*s* = 0, 1, 2,…, *S*) with *r* = 0, 1, 2, 3. Finally, we compared the distribution of *s* to the distribution generated by drawing three variables at random out of the ten used to generate each SDM. The probability of *r* = 0, 1, 2, 3 is given by

(6) $$P( {R = r} )\left\{ {\matrix{ {\matrix{ {0.29} \cr {\matrix{ {0.53} \cr {\matrix{ {0.175} \cr {0.008} \cr } } \cr } } \cr } } & {\matrix{ {\matrix{ {{\rm if}\; R = 0} \cr {\matrix{ {{\rm if}\; R = 1} \cr {{\rm if}\; R = 2} \cr } } \cr } } \cr {{\rm if}\; R = 3} \cr } } \cr } } \right.$$

We then used a one tailed Pearson’s chi-squared statistic to compare the expected and observed number of cases with zero, one, two, and three variables being correctly identified for all the 200 simulated species and for each weak and strong effect scenario separately (see Supplemental Information 2 for proof of how probabilities were derived).

### Case study: modeling *Toxostoma rufum*, the brown thrasher, in the continental US

Applications of ENMs to estimating species’ potential distributions remains an important part of the ecology literature, including ecology, conservation, and related fields such as disease or pathogen distributions. Across all of these uses, understanding variable contribution can assist on evaluating biological information within models and how those compare to real-world knowledge of species’ biology and ecology. To explicitly demonstrate the use of the new variable selection procedure, we provide a real-world case study for exploring the ecological requirements and distributions of the of the brown thrasher, *T. rufum*, a cosmopolitan bird species in the continental US. This species has been the focus of previous ENM papers, including some of the original studies applying GARP (Peterson, 2001).

#### Data

For this study, we used available data on the distribution of *T. rufum* from the Global Biodiversity Information Facility website (GBIF; https://www.gbif.org; accessed data: 30 October 2019). We downloaded all records of the species with corrected latitude and longitude pairs. Here, we limited our analysis to the continental US (lower 48 states). To further ensure data were correct identifications of the species, we downloaded a shapefile of the *T. rufum* density during the summer distribution from the US Geological Survey’s Breeding Bird Survey (BBS; https://www.mbr-pwrc.usgs.gov/bbs/bbs.html; accessed data: 30 October 2019). We clipped the BBS surface to the continental US. As other *Toxostoma* spp. occupy a similar ecological niche elsewhere in the continental US, we limited the GBIF occurrence data to only pixels within the clipped BBS raster. This resulted in 6,425 individual point locations. We then applied the *spthin* routine within the “spThin” R-package (Aiello-Lammens et al., 2015) with a 50 km buffer around each point to reduce effects of over sampling any portion of the range defined by the BBS raster layer. As GARP treats any pixel with at least one location as present, *T. rufum* locations were then sampled to spatially unique points based on the resolution of the environmental layers. For this analysis, we used 22 climatic and biophysical covariates as environmental coverages and all coverages were resampled to 2.5 arcminute (~ 4.5 × 4.5 km) resolution. Given the resolution of the environmental layers, the 6,425 point occurrences represented 657 unique pixel cells (~4.5 × 4.5 km) after thinning and running the spatially unique routine in the “GARPTools” R-package. Details of and data sources for environmental coverages are provided in Table 1.

**Table 1:** List of 22 environmental variables (coverages) used for *T. rufum* GARP experiment.

Environmental coverage (unit)	Names	Resolution	Source
Elevation (m)	Alt	1 km	WorldClim^a^
Bioclimatic data (°C or kg of water/kg of air)	Bio 1–19	2.5 arcminute	MERRAclim^b^
Mean NDVI (no unit)	wd0114a0	1 km	TALA^c^
NDVI annual amplitude (no unit)	wd0114a1	1 km	TALA

**Note:**

a The WorldClim elevation data were accessed from worldclim.org/ (Hijmans et al., 2005), which were derived from SRTM Elevation data.

b The MERRAclim dataset from the 2000s decade with the mean humidity version was downloaded from https://datadryad.org/(Vega, Pertierra & Olalla-Tárraga, 2016, 2017).

c NDVI measurements were accessed from the Trypanosomiasis and Land Use in Africa (TALA) research group (Oxford, United Kingdom; Hay et al., 2006); All the data were accessed on September 21, 2018.

#### Variable selection based on UI to predict *Toxostoma rufum*

**Figure 2:**
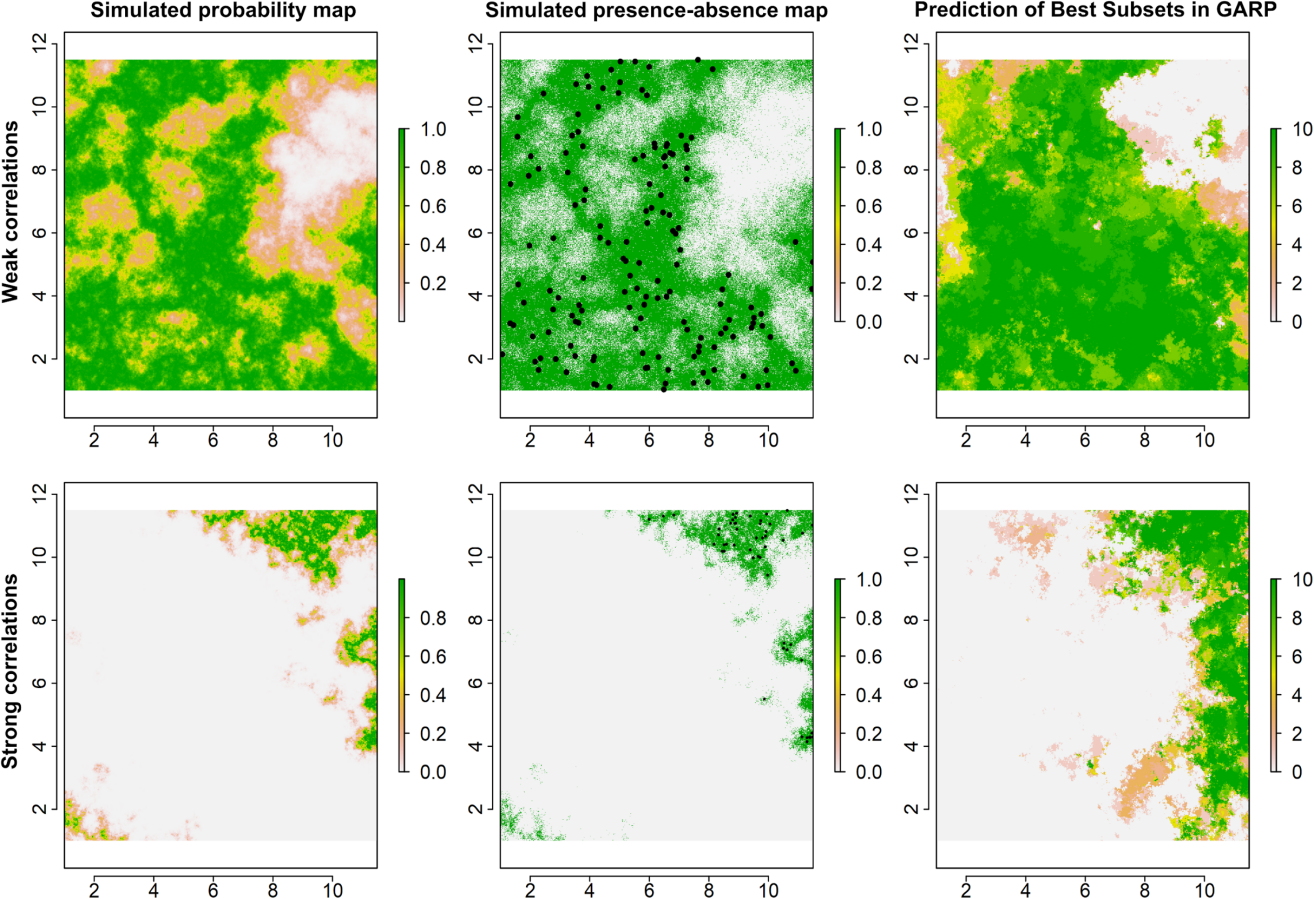
Simulated species distributions, occurrence (presence–absence) maps, and GARP prediction map for the best subset under the two scenarios where the correlation between species occurrence and environment are weak and strong. The black points are the presence locations extracted from occurrence map for modeling species distributions in GARP.

To explore the environmental coverages for *T. rufum*, we followed a similar procedure as for the simulated species. We first input all 23 environmental coverages in DekstopGARP. Since the true distribution of the species is unknown, and to evaluated the predicted distributions from GARP, we split the 657 spatially unique *T. rufum* occurrence locations into external training/testing with 75%/25% ratio prior to model construction. This allowed for 491 points to build models within GARP (which were then split again internally), and 166 points for post hoc model accuracy evaluation using GARPTools (Fig. 2). We built GARP experiments following the parameterization in Blackburn et al. (2007). In a first GARP experiment, we calculated the UI for each of the 23 variables and assumed them to be important if the UI value was smaller than 0.5. Finally, we re-ran the GARP experiment using only the variables identified to be important. We then repeated the analysis at UI values smaller than 0.25.

Predictive accuracies for the best subsets from the GARP experiments with the UI-based reduced variable sets was evaluated using a combination of AUC, omission, and commission rates based on the external testing dataset (Lim & Klein, 2006; Peterson, Papeş & Eaton, 2007). The AUC, although not an ideal metric for accuracy estimation (Lobo, Jiménez-Valverde & Real, 2008), is useful to identify models that perform well (Hanley & McNeil, 1982; Mullins et al., 2013; Sloyer et al., 2018). The 10-model best subset from the UI-based experiments was summated to map the potential geographic distribution of *T. rufum* for the continental US. We compared UI-limited distributions to the distribution with all variables included. All data and R code for simulating species’ distributions for this study are available for download and use (https://github.com/jkblackburn/Trufum_GARPTools).

## Results

### Simulated species and variable selection performance in simulation scenarios

**Figure 3:**
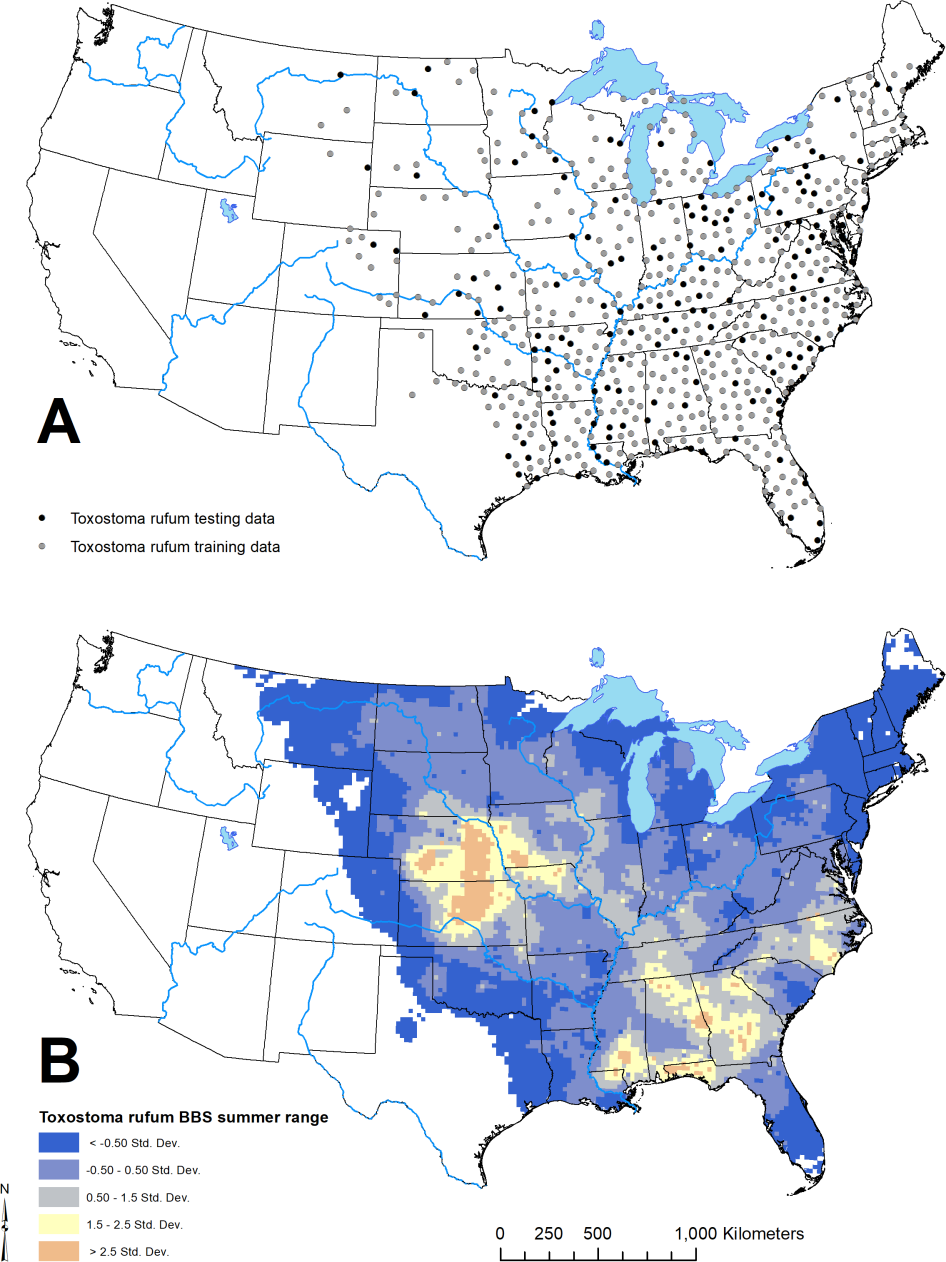
Geographic distribution of points representing *Toxostoma rufum*, the brown thrasher—a cosmopolitan bird species, used for training and testing the GARP model experiment in the case study (A); estimate of the breeding bird survey (BBS) extent for the species in the continental US used to limit occurrence points downloaded from the Global Biodiversity Information Facility (B).

Examples for the probability maps of species distributions, binary occurrence maps simulated with weak and strong correlations, and GARP predictions based on those simulated species are illustrated in Fig. 3. We found that UI and GARP performed well during the simulations. For the 200 simulated species we found that the observed number of species with *r* = 0, 1, 2, 3 does not follow the distribution of random draws (χ^2^ = 724.3, *n* = 200, df = 3, *p* < 0.0001) and in particular the observed number of species with *r* = 2 and *r* = 3 is significantly higher than expected by chance (Table 2). We found a similar result when analyzing separately the species in which environmental covariates were assumed to have a weak and strong effect on the geographic distribution (Table 2; weak: χ^2^ = 367.2, *n* = 100, df = 3, *p* < 0.0001; strong: χ^2^ = 360.1, *n* = 100, df = 3, *p* < 0.0001). Finally, we found no differences in the observed number of species with *r* = 0, 1, 2, 3, when comparing the species simulated using strong and weak coefficients (χ^2^ = 2.64, df = 3, *p* = 0.45).

**Table 2:** Summary of the observed and expected number of species for which the variable selection method correctly identified zero, one, two or three out of three variables used to simulate the species distribution. The counts are tallied for 200 simulated species (All) and separated by the 100 species for which we selected Weak and Strong influence of the environmental variables on determining the species distribution.

Scenarios	0		1		2		3	
	Observed	Expected	Observed	Expected	Observed	Expected	Observed	Expected
Weak	4	29	24	53	57	17	15	1
Strong	9	29	26	53	49	17	16	1
All	13	58	50	105	106	35	31	2

### Geographic distribution and ecological requirements of *T. rufum*

**Figure 4:**
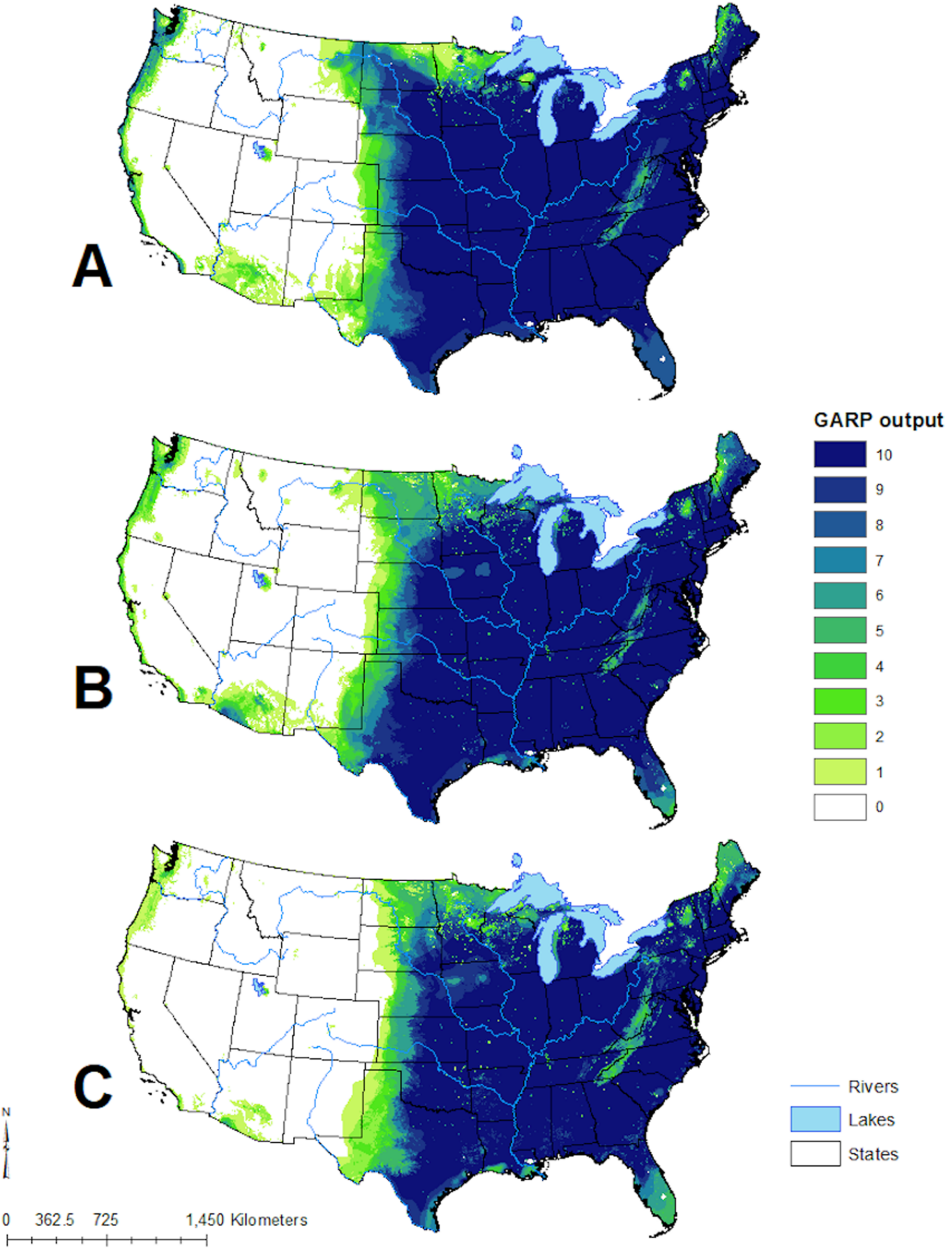
Predicted geographic distribution of *Toxostoma rufum*, the brown thrasher, in the continental US based on three different niche modeling experiments. (A) All 22 variables used in the study were included in the prediction; (B) prediction based on a reduced set of 13 variables based on a UI threshold of 0.5; (C) prediction based on a reduced set of 10 variables based on a UF threshold of 0.25. Darker shades of blue reflect greater model agreement between individual models in the best subset or greater confidence in the prediction of presence of the species in those pixels.

**Figure 5:**
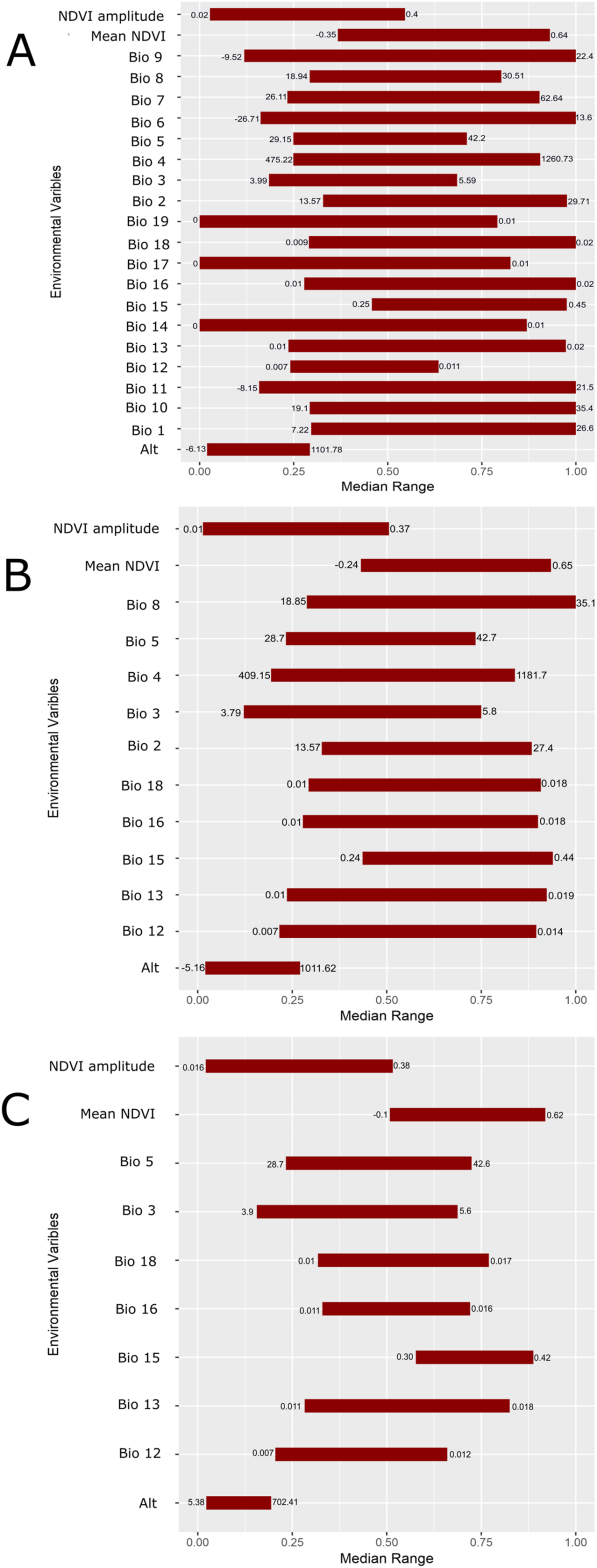
Scaled median range of the covariates from the best subset of each GARP experiment to predict the distribution of *Toxostoma rufum* in the continental US using all variables (A), those selected with the the UI set to 0.5 (B), and those variables included with UI set to 0.25 (C). Variables were scaled from 0 to 1 for all covariates. Those with narrower ranges have a greater influence on the predicted distribution in the models. The values on the bars show the actual range of the environmental conditions based on that ecological niche modeling experiment.

The geographic distributions of all three experiments are illustrated in Fig. 4. Applying the UI tool, we selected 13 of the original 22 variables with UI less than 0.5, including the climatic (temperature and moisture) seasonality, elevation, mean NDVI, and seasonality of NDVI (Fig. 5). AUC values, omission, and commission of the GARP experiments are reported in Table 3. The UI 0.5 reduced variable set was 0.756, with a total omission of 0.012 and an average omission of 5.97%, indicating high predictive accuracy of test points; AUC values of cosmopolitan species are often below 0.8 on large landscapes due to the large area predicted present (McNyset, 2005). When we applied the UI cutoff of 0.25, only 10 variables were included and the AUC value was 0.767, with total omission 0.024% and average omission 8.1%. Between UI 0.5 and 0.25, the total omission was slightly lower in UI 0.5. In both cases, the overall model accuracy was good, and the models became more parsimonious, making it easier to evaluate the contribution of each variable to model prediction. There was an overall improvement in AUC values from all variables to UI 0.25, so models performed better (at least slightly) as the total numbers of variables were reduced.

**Table 3:** Accuracy metrics of GARP experiments to model *Toxostoma rufum*, the Brown Thrasher, across the continental US (lower 48 states) based on three experiments. All variables (23 total variables, no UI calculated), UI 0.5 threshold (UI less than 0.5) and UI 0.25 threshold (a more conservative cutoff). Models were performed with the same parameter setting each time, selecting only variables in the accompanying plots in Fig. 5.

GARP experiment	Total omission	Average omission	Total commission	Average commission	AUC	AUC Standard Error	AUC Z-score
Tr50* All variables	0.03	10.9	41.46	55.44	0.745	0.02	15.7
Tr50 UI 0.5 threshold	0.012	5.97	39.73	53.01	0.756	0.02	15.7
Tr50 UI 0.25 threshold	0.024	8.1	34.69	48.22	0.767	0.02	14.9

**Note:**

* *Toxostoma rufum* locations based on 50 km thinning using spThin R package.

## Discussion

In this study, we present a new variable selection rubric for the GARP ecological niche modeling system based on prevalence rates and median ranges of the variables in the dominant presence rules in best subsets. Overall, the variable selection methodology performed well by identifying the important ecological variables defining the distribution of the simulated species. We found a high probability of identifying all or most of the variables that are important to the distributions of those species, irrespective of the relative influence of the variables on determining the distribution. In over 65% of the cases, our UI correctly identified at least two of the three variables defining the species environmental envelope. In the real-world case study, we identified that 13 of 22 were of high importance using a UI threshold of 0.5 and 10 of 22 were included at a 0.25 cutoff in determining the distribution of *T. rufum*, the brown thrasher, a bird species with a cosmopolitan distribution in the US. The important variables included temperature and moisture seasonality, altitude, and vegetation index.

Our new methodology for estimating variable contribution in GARP was developed considering the explanatory power within a modeling experiment measured by the frequencies the variables are used and the biological information within the experiment using those variables. The explanatory power of the variables here were first measured by the number of times that the variables were selected to predict the presence of the species in the best subsets. This idea follows from the estimation of variable contributions in some machine learning algorithms, such as Boosted Regression Trees (BRTs) and random forests, which calculate the variable contributions based on the number of times the variable is used to split the trees (Friedman & Meulman, 2003). Additionally, the biological information within the GARP experiment was quantified by the median ranges of the variables. Variables with a narrow range of values that will predict the presence of the species suggest species distributions are sensitive to those conditions (Mullins et al., 2011). Those variables might have a higher explanatory power as they may restrict the species distribution in both ecological and geographical space. If a species has a wide tolerance to a specific variable, then this variable may necessarily have low explanatory power at least in the geographic area considered. Variables that are identified with less contributions to the model could also be important conditions for the species survival but allow a species to be widespread or are not the common requirement across the population of occurrences. UI considering both the frequency the variable used to predict species presence and biological information would help identify common conditions confining a species’ distribution, which could be used to infer the underlying biological mechanisms of species survival.

We tested the performance of the proposed variable contribution estimation method in simulated species with both weak and strong correlations between species occurrence and environmental covariates and found overall good performance. Our generation of the simulated species, although is simpler than reality, follows an ecologically realistic scenario in which species distributions are a function of multiple factors and respond to the environment under a bell curve determined by these covariates and is not limited to one type of species (Elith & Leathwick, 2009). It is noteworthy that the simulator developed for this study is available for use and is not specific to any ENM approach, allowing researchers to fit this methodology to other ENMs. The test of the performance of UI in different simulation scenarios evaluates its general ability of correctly identifying the primary covariates that contribute to species distributions. We found that the majority of the cases in both simulation scenarios selected most (2/3 or all three) variables correctly, which indicates that our variable selection method performs well regardless of the strength of the environment in determining the species distribution. Overall, the good performance of UI indicates that this method allows the identification of the environmental variables that are important in defining a species distribution, and thus can allow for inferences about the physiological tolerances of the species and the dispersal abilities across a landscape.

The incorporation of the optimal variables in the model is important for making inferences about the ecology and the mechanisms determining species distributions. Including the optimal set of variables in the SDMs could increase the model accuracy and provide a better understanding of the ecological requirements for species survival. Also, filtering the most useful variables among a series of candidate variables might help to reduce noise in the predictions. In the real-world case study, we show that variable selection using our UI tool resulted in model improvement while reducing the models to a more parsimonious coverage set. The reasonable AUCs, and overall low omission values (indicating accurate prediction of holdout data) of GARP outputs for *T. rufum* indicated a good performance of the modeling system with the selected optimal variable sets.

The distribution of *T. rufum* predicted here with the reduced variable sets was in line with the BBS estimates; minor areas of prediction in the west are known to be areas filled by sister taxa (Peterson, 2001). Overall, we illustrate with a real world example that these tools can be used to select parsimonious environmental coverages to predict species distributions, which may be helpful identifying ideal variables sets for species of interest. Additionally, this variable contribution estimation approach has recently been applied to understand genetic-environmental associations of the *Bacillus anthracis* A1. a sub-lineage and predict its geographical distribution in the continental US as a proxy for anthrax risk for this strain (Yang et al., 2020).

## Conclusions

The method described herein presents a procedure of evaluating variable contributions based on median range and the frequency of the variable used to predict the presence of the species. This variable contribution estimation procedure was employed using GARP system, but the idea of the consideration of both the explanatory power and environmental coverage when selecting variable is highlighted and is applicable to other SDMs. The new variable selection method was tested via simulations which we found to be accurate in the identification of the important environmental variables in determining the distribution of simulated species. We employed this method to understand the ecological requirements and geographic distribution of *Toxostoma rufum—*a bird species modeled in early ENM studies. The optimal ecological coverages selected by the variable selection method include the seasonality of temperature and moisture, elevation, mean and seasonality of NDVI. The predicted distributions were primarily restricted to central and eastern US. The variable selection idea presented here provides an objective way to identify the variables that are most important for predicting species distributions with GARP, which is analogous to the variable selection methods integrated in other SDM algorithms (e.g., Maxent or BRTs) and fills the gap in the practical application in the estimation of variable contributions and variable selections in GARP.

## Supplemental Information

10.7717/peerj.8968/supp-1 Supplemental Information 1 Simulated environmental layers with an extent of 10.5 × 10.5 degree and 0.01 × 0.01 degree resolution; the origins of both *x* and *y* coordinates start from 1.

10.7717/peerj.8968/supp-2 Supplemental Information 2 Derivation of the probabilities *r* = 0, 1, 2, 3 based on a random draw.

10.7717/peerj.8968/supp-3 Supplemental Information 3 Supplemental R code for Simulating Species Distributions.

## Additional Information and Declarations

### Data Availability

The following information was supplied regarding data availability: Code and simulated data for this study are available at: https://github.com/cghaase/GARPTools. The DesktopGARP DG v1.1.3 described in the methods is available for download: https://github.com/jkblackburn/DesktopGARP1.1.3. The data from the case study of *T. rufum* and the R code for simulating species distributions is available at: https://github.com/jkblackburn/Trufum_GARPTools.
