# Supplementary figures and images for: Exploring environmental coverages of species: a new variable contribution estimation methodology for rulesets from the genetic algorithm for rule-set prediction
Source: PeerJ. 2020 May 12;8:e8968. doi: 10.7717/peerj.8968 (PMC7227675; doi:10.7717/peerj.8968)

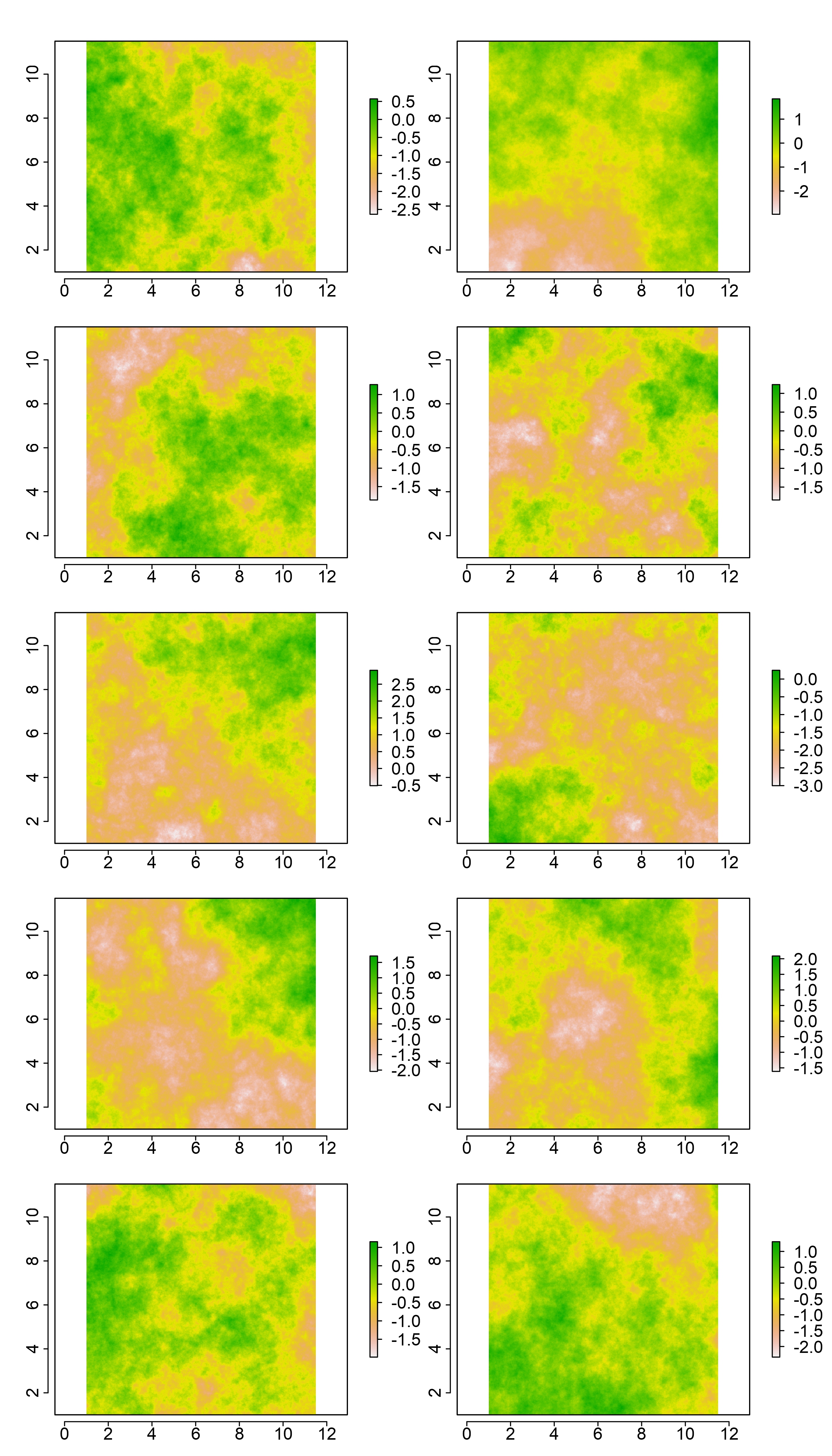

Supplement: Supplemental Information 1 [file peerj-08-8968-s001.png]
