# Supplementary material for: Exploring environmental coverages of species: a new variable contribution estimation methodology for rulesets from the genetic algorithm for rule-set prediction
Source: PeerJ. 2020 May 12;8:e8968. doi: 10.7717/peerj.8968 (PMC7227675; doi:10.7717/peerj.8968)
Supplement: Supplemental Information 2 [file peerj-08-8968-s002.docx]

Derivation of the probabilities *r =* 0, 1, 2, 3 based on a random draw.

For *r =* 0, the probability is given by the joint probability of obtaining an incorrect variable in each of the three draws. In the first draw, there are 7 out of 10 variables that were not used to generate the species distribution thus, the probability of choosing an incorrect variable in the first draw is 7/10. Then, in the second draw, there are only 9 variables left to choose from and only 6 of them are incorrect such that the probability of obtaining an incorrect variable in the second draw is 6/9. Using the same rationale, the probability of choosing an incorrect variable in the third draw is 5/8. Thus, the probability of picking 3 incorrect variables out of 10 possible ones without replacement is just the multiplication of 7/10, 6/9 and 5/8. Thus $P\left( r=0 \right)=(7/10)*(6/9)*(5/8)\approx0.3$. Now, for *r =* 3, using the same rationale as for *r =* 1, for the first draw there are three correct variables out of ten, in the second draw five that we chose a correct variable in the first draw there are only two out of nine left and in the third draw, given that we chose correctly the variables in the first and second draws there is only one correct variable out of eight to choose from. Thus $P\left( r=3 \right)=(3/10)*(2/9)*(1/8)\approx0.008$. For the cases in *r =*1 and *r =*2 we need to take into account the order in which we can draw one or two correct variables. For example, for *r =* 1, we can choose the correct variable in the first, second or third draw. This means that we have three ways of choosing one variable out of ten. It is, that in the first draw we choose the correct variable and in the other two are incorrect or that we choose an incorrect variable in the first draw, the correct one in the second and an incorrect one in the third again or that we choose two incorrect variables in the first two draws and a correct one in the third draw. Let C be the draw of a correct variable and I be the draw of an incorrect variable. Thus, the chances of getting exactly one correct variable out of ten in three draws is represented by, CII, ICI, IIC. This is, $P\left( r=1 \right)=(3/10)*(6/9)*(5/8)+(7/10)*(3/9)*(6/8)+(7/10)*(6/9)*(3/8)=0.525$. Similarly, for *r =*2, we have that the ways of picking two correct variables out of ten are, CCI, CIC, ICC. $P\left( r=2 \right)=(3/10)*(2/9)*(7/8)+(3/10)*(7/9)*(2/8)+(7/10)*(3/9)*(2/8)=0.175$. Since the random variable *R* can only take values of 0, 1, 2, 3, the sum of the probabilities must add up to one. $P\left( R=r \right)=P\left( r=0 \right)+P\left( r=1 \right)+P\left( r=2 \right)+P\left( r=3 \right)=0.291+0.525+0.175+0.008\approx1$. Because of the precision with which we are defining the probabilities, the latter does not add up to one but taking into account all decimal places it does.
